# Expulsion of Tænia by Pumpkin Seeds

**Published:** 1856-03

**Authors:** W. W. Ely

**Affiliations:** Rochester, N. Y.


*Expulsion of Tænia by Pumpkin Seeds.*—I am indebted to Dr. H. B. Sherman, of this city, for the following case:—A child, aged eighteen months, was presented to him for advice, having glandular disease of the neck, tumid abdomen, unhealthy countenance, and symptoms which led him to suspect the existence of tape worm. This impression was confirmed by seeing fragments of the worm which had been obtained from the fœcal discharges. He accordingly prepared a gill of emulsion from two ounces of pumpkin seeds, which the child took on the 24th of January, 1855, followed after three hours with castor oil. In two hours more, a tape worm was discharged, measuring full fifteen feet in length. At the time of this report, a few weeks since, the child was in excellent health, with no signs of a return of the verminous disorder.

Miss W. applied to me in December last to be treated for tape worm. On the 30th of December, at 5 o'clock, A. M., she took eight ounces of pumpkin seed emulsion, and in three hours after she had three tablespoonfuls of castor oil. The medicine operated between 3 and 4 o'clock, P. M. The worm was voided in the first operation, and measured eighteen and a half feet in length. A few days after, the remedy was repeated, as an experiment, but no further indications of tænia were obtained.

The advantages of the method employed in the above cases are obvious. It is simple, mild and efficacious. To avoid disappointment in prescribing for tape worm, a few points must be attended to. Patients are often suspected of having tape worm from subjective symptoms only. These are not sufficient, and the failure of a remedy in such cases is presumptive proof that the diagnosis was wrong. The physician should *in all cases* insist upon ocular demonstration, which can easily be obtained, since portions of the worm are habitually voided by those who are infested with this parasite. The medicine also should be properly prepared and administered. For the convenience of those who have not a formula at hand, the following directions may be of service. Bruise three ounces of pumpkin seeds thoroughly in a mortar; add cold water, and beat the seeds with it intimately, until by expression and straining they yield eight ounces of emulsion. Let the patient take the above quantity in the morning, fasting, and follow it in two or three hours with a full cathartic dose of castor oil. Cold water is to be allowed, if desired, as a beverage, but no food should be taken, until after the operation of the purgative.—*Boston Med. and Surg. Journal.* W. W. Ely.

Rochester, N. Y., Jan. 19th, 1856.

